# Performance of Qure.ai automatic classifiers against a large annotated database of patients with diverse forms of tuberculosis

**DOI:** 10.1371/journal.pone.0224445

**Published:** 2020-01-24

**Authors:** Eric Engle, Andrei Gabrielian, Alyssa Long, Darrell E. Hurt, Alex Rosenthal

**Affiliations:** Office of Cyber Infrastructure & Computational Biology, National Institute of Allergy and Infectious Disease, National Institutes of Health, Bethesda, MD, United States of America; Duke University Hospital, UNITED STATES

## Abstract

Availability of trained radiologists for fast processing of CXRs in regions burdened with tuberculosis always has been a challenge, affecting both timely diagnosis and patient monitoring. The paucity of annotated images of lungs of TB patients hampers attempts to apply data-oriented algorithms for research and clinical practices. The TB Portals Program database (TBPP, https://TBPortals.niaid.nih.gov) is a global collaboration curating a large collection of the most dangerous, hard-to-cure drug-resistant tuberculosis (DR-TB) patient cases. TBPP, with 1,179 (83%) DR-TB patient cases, is a unique collection that is well positioned as a testing ground for deep learning classifiers. As of January 2019, the TBPP database contains 1,538 CXRs, of which 346 (22.5%) are annotated by a radiologist and 104 (6.7%) by a pulmonologist–leaving 1,088 (70.7%) CXRs without annotations. The Qure.ai qXR artificial intelligence automated CXR interpretation tool, was blind-tested on the 346 radiologist-annotated CXRs from the TBPP database. Qure.ai qXR CXR predictions for cavity, nodule, pleural effusion, hilar lymphadenopathy was successfully matching human expert annotations. In addition, we tested the 12 Qure.ai classifiers to find whether they correlate with treatment success (information provided by treating physicians). Ten descriptors were found as significant: abnormal CXR (p = 0.0005), pleural effusion (p = 0.048), nodule (p = 0.0004), hilar lymphadenopathy (p = 0.0038), cavity (p = 0.0002), opacity (p = 0.0006), atelectasis (p = 0.0074), consolidation (p = 0.0004), indicator of TB disease (p = < .0001), and fibrosis (p = < .0001). We conclude that applying fully automated Qure.ai CXR analysis tool is useful for fast, accurate, uniform, large-scale CXR annotation assistance, as it performed well even for DR-TB cases that were not used for initial training. Testing artificial intelligence algorithms (encapsulating both machine learning and deep learning classifiers) on diverse data collections, such as TBPP, is critically important toward progressing to clinically adopted automatic assistants for medical data analysis.

## Introduction

Tuberculosis (TB) caused over 1.6 million deaths in 2017. The global treatment success rate was 82% in 2016, and for drug-resistant TB (DR-TB), the rate was 55%. In addition, the WHO estimated 10 million TB cases during 2016 combined with a 64% TB treatment coverage rate, (notified / estimated incidence) [1], thus approximately 3.6 million TB cases were not diagnosed and treated. Current TB screening methods include the CXR, and it remains in widespread clinical use providing high sensitivity as a diagnostic tool and additional insight into TB disease prognosis [2, 3].

The value of the CXR in early TB detection is well documented [2, 3]. CXR has the best sensitivity of all clinical tests in the range of 87% to 98% [2]. This compares well with the sensitivity of conventional microscopy range from 32% to 94%, fluorescence microscopy in range from 52% to 97% [4], and sensitivity of tuberculosis diagnostics Xpert Ultra and Xpert of 63% and 46% respectively [5].) In addition, the CXR has diagnostic utility beyond TB, and it remains a front-line tool for assessing and diagnosing a wide range of health issues [3,6].

Yet a shortage of radiologists limits its usage in low-resource disadvantaged populations with a high burden of TB. For many, visiting the clinic for radiological screening is relatively expensive [7]. The challenges with the CXR also include a lack of globally accepted reading standards and inter- and intra-rating issues [8, 9, 10]. These problems impact both TB diagnostics and TB monitoring. TB diagnostics are addressed with a single TB or not TB disease classifier. TB monitoring requires a more complete set of TB specific CXR annotation classifiers.

Specific to TB diagnostics, in attempt to remedy this situation, several countries reported CXR screening programs in rural settings with X-ray machines mounted in mobile vans and outfitted with implementations of machine learning and deep learning classifiers that are performing quick automated TB screening [11,12]. As this practice is adopted in more settings, the need for highly efficient predictive methods cataloging additional lung features is clear.

The Food and Drug Administration recently approved the first artificial intelligence powered X-ray device to scan images and detect pneumothorax [13]. Artificial intelligence deep learning methods are moving from the setting of compelling technical research and development projects, to use in clinical research, to mainstream medical practice.

The pathway to create and test these methods is to collect a large volume of representative data that contains a sufficient number of specific types of each abnormality. Data are then separated into sets for training, validation and testing. The quality of the data used for creating the classifier is directly related to the observed results when the classifier is used against subsequent novel input data.

TB specific classifiers are available via two commercial entities–Qure.ai qXR product (http://qure.ai/qxr/) and Delft Imaging Systems CAD4TB (https://www.delft.care/cad4tb/)—and numerous additional models can be found in the literature [14–19]. Qure.ai qXR product (http://qure.ai/qxr/) provides a commercially available set of software CXR image classifiers, and they have made this service available to the TB Portals Program.

Details of the Qure.ai deep learning classifiers are proprietary information; however, a broad overview is available in a pre-print article, and the company’s web site. The Qure.ai classifiers were developed on 1.2 million CXRs obtained from hospitals using 22 X-ray machine models from 6 vendors. Since the images varied in resolution and quality, they were down-sampled and re-sized to a standard. During this processing several abnormality-specific data augmentation techniques were applied to the input CXR images. Convolutional neural networks (CNNs)–specifically Qure.ai modified versions of densenets and resnets open source algorithms—were trained to identify 12 individual CXR abnormalities. A pre-training process that focused on separating chest X-rays from other medical X-rays was used by Qure.ai rather than selecting an open source pre-trained network. Qure.ai trained multiple models to detect each abnormality. A subset of the models was selected using appropriate heuristics and a majority ensembling schedule was used to combine these models and score the presence or absence of a specific abnormality [20–22].

On independent sets of CXRs that were not used in the training, Qure.ai reports an update of the March 2018 reported results [20] with TB diagnostic accuracy measured as the receiver operating characteristic area under the curve of 97% against the publicly available Montgomery County dataset and 95% against the Shenzhen Hospital imaging dataset. An additional study of the Qure.ai CXR lung feature classifiers yielded promising results. This study drew 874 CXRs randomly from the ChestX-ray8 database downloaded from the National Institute of Health website (https://nihcc.app.box.com/v/ChestXray-NIHCC, accessed on January 30, 2018) [23].

However, all the datasets noted above have a limited number of CXRs from patients with DR-TB. Hence, we decided to test the performance of Qure.ai classifiers on NIAID TB Portals collection of CXRs and CTs, containing multiple cases of DR-TB, to ensure the efficiency of classifiers in annotating these most dangerous and hard to treat instances.

Several non-TB specific CXR databases and radiograph classifiers are extant. These include CheXpert (224,316 CXR images) and the associated labeler [24], as well as those images released in the ChestX-ray8 database (112,120 CXRs) by the NIH Clinical Center [25]. The challenge with using these images for training and testing classifiers is that there is a low proportion of TB disease specific images, and lower percentage of DR-TB images with associated annotations and clinical data [24–26].

In 2012, the NIAID TB Portals Program (TBPP) [27] initiated the development of a novel data repository containing socioeconomic, geographic, clinical, laboratory, radiological, and pathogen genomic information from deidentified patient cases. This TBPP initiative brings disparate, local, clinical records of TB cases, with an emphasis on DR-TB cases, from countries burdened with TB to the attention of the global research community in the form of an open-access online resource.

As of January 2019, TBPP has made publicly available 1,425 physician-validated patient cases from ten country sites (Azerbaijan, Belarus, Moldova, Georgia, Romania, China, India, Kazakhstan, South Africa and Republic of Congo), 1,179 (83%) of which are DR-TB and 614 (43%), 669 (47%), and 754 (53%) of which contain CXR, CT, and genomic data, respectively. With the TBPP emphasis on DR-TB (1,179 patient cases), the database is a unique and valuable resource for the examination of the most rare, atypical, and dangerous TB patient cases. For all patient cases, 410 (29%) contain both CXR and CT images. Currently, of the 1,538 CXR images stored within TBPP data, 346 CXR images are annotated by a single attending radiologist.

One of TBPP goals is to utilize its unique data resource to identify and test promising computer-generated models in the areas of TB imaging and genomics. Bringing these models forward to empower researchers, health care professionals, and informaticians toward the development of novel diagnostics and personalized treatment regimens.

In this paper we report the results of comparison of the radiologist annotations for CXRs and known patient case outcomes with the predicted Qure.ai deep learning classifier scores.

## Materials and methods

The TBPP database is a de-identified, curated, physician validated, and accessible resource (https://Data.TBPortals.niaid.nih.gov). The origins and contents of the TBPP database is fully described in Rosenthal et al [27]. Over 170 data fields from socioeconomic, geographic, clinical, laboratory, radiological, and pathogen genomic information are linked via the patient case identifier.

This analysis originates from a focus on the 1,538 CXR images and 666 CT images stored within TBPP data, of which 346 CXR images and 184 CT images are annotated by the same single attending radiologist from the Republican Scientific and Practical Centre for Pulmonology and Tuberculosis, Minsk, Republic of Belarus. The annotations were created and captured by a single radiologist using a pre-defined collection form and data entry tool.

During December of 2018, TBPP CXRs were provided to Qure.ai for processing. Of the CXR raster images provided to Qure.ai, 89% measured 1,800 by 1,800 pixels. All images were 200 pixels per inch. Lossless, an 8-bit grey-scale image format, is used for storing 69% of the files with 31% being kept in the a three-channel 24-bit format.

Qure.ai provides the TB classifiers output as a free service for research purposes in collaboration with the TBPP. The results for this analysis were provided during December 2018. As shown in Table 1, Qure.ai provides a set of 12 deep learning classifier scores for the following common CXR findings.

**Table 1:** Qure.ai qXR (http://qure.ai/qxr/) TB deep learning classifiers.

Abnormal	Indication of abnormality on the chest X-ray
Blunted CP angle	Indicating the presence of a pleural effusion or pleural thickening
Cardiomegaly	Increased heart size, increased cardio-thoracic ratio
Pleural Effusion	A build-up of excess fluid between layers of pleura outside of the lungs
Nodule	Small well-defined opacity in the lung fields
Hilar lymphadenopathy	Hilar enlargement, prominence or visible lymph nodes
Cavity	A gas-filled space, seen as a lucency or low-attenuation area
Opacity	A general term indicating an abnormal radiopaque region, includes a wide range of well circumscribed or ill-defined abnormalities in the lung fields
Atelectasis	Decrease in lung capacity
Consolidation	Airspace opacification, often following a lobar pattern
Tuberculosis	Indication of TB disease on the chest X-ray
Fibrosis	Reticular shadowing or evidence of scarring

Among these 12 Qure.ai classifier findings, five were in common with TBPP CXR and CT annotations: cavity, nodule, atelectasis, pleural effusion, hilar lymphadenopathy. The classifier outcomes consist of a continuous score in the range of zero to one. A threshold is set by Qure.ai that defaults to more sensitivity and less specificity (along the ROC curve), and a binary present/absent prediction is indicated as well. In practice this setting is adjusted during deployment in accordance with the screening, radiological workflow prioritization, or research needs. We used the default.

The plan for testing Qure.ai classifiers on the data from TBPP database was as follows.

- Comparison of TBPP radiologist annotated CXRs features with features predicted by Qure.ai for the same CXRs
- Validating, with the help of TBPP radiologist annotated CTs whether the features found on CXRs and predicted by Qure.ai are consistent
- Using TBPP clinical information regarding patient case treatment outcome, seek most statistically significant correlations with Qure.ai classifiers

The first radiologist-annotated reference standard comparison cohort consists of 346 CXRs from 311 patient cases. Considering the number of TBPP CXRs that did not have the radiologist’s annotations we sought to exploit the TBPP CT images that were annotated. CT annotated images that occurred within 30 days of a CXR from the same patient case were matched. This identified 184 CT images from 137 patient cases–a second independent radiologist-annotated reference standard. This comparison set is of interest because the CT images provide the Radiologist with much more information with which to identify the specific patient lung features. Examining patient case outcome, we identify our two cohorts for analysis: 220 patients with an outcome of cured, and 61 with an outcome of died or treatment failure.

The present/absent indications were compared using Fisher’s exact test. The continuous score indication was evaluated using Wilcoxon-Mann-Whitney test (WMW) and receiver operating characteristic (ROC) curves. As a nonparametric test, WMW was selected because it does not depend on a normal data distribution for a test of the null hypothesis that it is equally likely that a randomly selected score from one cohort will be less than or greater than a randomly selected score from the other cohort. ROC curve analysis will provide an overall estimate of accuracy with a confidence interval. All analysis results were generated using SAS/STAT software 14.1, SAS Software version 9.4 of the SAS System for Windows Workstation Copyright © 2002–2012 by SAS Institute Inc.

## Results

The first TBPP reference standard cohort consists of 346 annotated CXRs from 311 patients. Among these cases there are 85(24.6%) extensively drug resistant, 186(53.7%) MDR or mono drug resistant, and 75(21.7%) drug sensitive. The patient case definition distribution is 187(54.1%) new, 79(22.8%) failure, 63(18.2%) relapse, 9(2.6%) treatment after default, and 8(2.3%) other. The patient case outcomes are 179(51.7%) cured, 84(24.3%) completed, 29(8.4%) default, 25(7.2%) failure, 19(5.5%) died, and 10(2.9%) unknown.

TBPP annotations for the 346 CXRs indicate the presence of hilar lymphadenopathy (29, 8.4%), cavity (84, 24.3%), atelectasis (335, 96.8%), pleural effusion (35, 10.1%), and nodule (315, 91%). We found statistically significant correspondence between human-provided and deep learning-based measures for hilar lymphadenopathy, cavity, pleural effusion, and nodule. The summary statistics in comparison to the Qure.ai binary prediction score is shown in Table 2.

**Table 2:** 346 TBPP CXR Annotations versus Qure.ai Binary Prediction, Crosstabulation Summary Statistics.

Lung Feature	CXR Accuracy	P-Value	Sensitivity	Upper CL	Lower CL	Specificity	Upper CL	Lower CL
**Hilar lymphadenopathy**	73%	<0.0001	62.1%	79.7%	44.4%	74.1%	69.3%	79.0%
**Cavity**	80%	<0.0001	75.0%	84.3%	65.7%	82.1%	77.4%	86.7%
**Atelectasis**	21%	0.5181	19.4%	23.6%	15.2%	72.7%	46.4%	99.0%
**Pleural Effusion**	91%	<0.0001	60.0%	76.2%	43.8%	94.9%	92.4%	97.3%
**Nodule**	61%	0.0003	59.7%	65.1%	54.3%	74.2%	58.8%	89.6%

The second TBPP reference standard cohort consists of 184 CT images from 137 patient cases. The resistance distribution among these cases is 47(25.5%) extensively drug resistant, 85(46.2%) MDR or mono drug resistant, and 52(28.3%) drug sensitive. The patient case definition distribution is 115(62.5%) new, 41(22.3%) failure, 22(12%) relapse, 2(1%) treatment after default, and 4(2.2%) other. The patient case outcomes are 80(43.5%) cured, 40(21.7%) completed, 10(5.4%) default, 18(9.8%) failure, 22(12%) died, and 14(7.6%) unknown. There were no cases with a defined outcome of still on treatment.

TBPP annotations for the 184 CTs indicate the presence of hilar lymphadenopathy (39, 21.2%), cavity (95, 51.6%), atelectasis (78, 42.4%), pleural effusion (133, 72.3%), and nodule (23, 12.5%). The summary statistics in comparison to the Qure.ai binary prediction score is shown in Table 3. Again, we found a statistically significant correspondence between expert annotations and deep learning-based predictions for hilar lymphadenopathy, cavity, atelectasis, and pleural effusion.

**Table 3:** 184 TBPP CT Annotations versus Qure.ai Binary Prediction, Crosstabulation Summary Statistics.

Lung Feature	Imputed CT Accuracy	P-Value	Sensitivity	Upper CL	Lower CL	Specificity	Upper CL	Lower CL
**Hilar lymphadenopathy**	65%	0.0318	69.7%	77.1%	62.2%	48.7%	33.0%	64.4%
**Cavity**	65%	<0.0001	84.3%	91.8%	76.7%	46.3%	36.3%	56.3%
**Atelectasis**	72%	<0.0001	89.6%	95.4%	83.8%	47.4%	36.4%	58.5%
**Pleural Effusion**	42%	0.0214	92.2%	99.5%	84.8%	22.6%	15.5%	29.7%
**Nodule**	45%	0.1629	45.3%	53.0%	37.7%	39.1%	19.2%	59.1%

Additional comparisons of 346 TBPP CXR annotations versus Qure.ai classifiers were completed. We examined the accuracy for 271 drug-resistant (Table 4) and 75 drug-sensitive (Table 5) TB patient cases with CXR. Considering these are distinct patient groupings commonly used in TB research, we note the difference in accuracy for the hilar lymphadenopathy annotation between the groups. The relationship is statistically significant for drug resistant TB (p<0.0001) with 20 positive radiologist annotations, but not for those CXR from patient cases that are sensitive to TB drugs (p = 0.3557) for 9 positive radiologist annotations.

**Table 4:** 271 TBPP CXR Annotations for Drug Resistant TB versus Qure.ai Binary Prediction, Crosstabulation Summary Statistics.

Lung Feature	CXR Accuracy	P-Value	Sensitivity	Upper CL	Lower CL	Specificity	Upper CL	Lower CL
**Hilar lymphadenopathy**	77%	<0.0001	65.0%	85.9%	44.1%	77.7%	72.5%	82.8%
**Cavity**	80%	<0.0001	75.6%	85.2%	66.1%	82.4%	77.0%	87.8%
**Atelectasis**	22%	0.737	20.2%	25.0%	15.3%	75.0%	45.0%	100.0%
**Pleural Effusion**	91%	<0.0001	62.1%	79.7%	44.4%	94.6%	91.8%	97.5%
**Nodule**	62%	0.0046	60.9%	67.0%	54.8%	69.6%	50.8%	88.4%

**Table 5:** 75 TBPP CXR Annotations for Sensitive TB versus Qure.ai Binary Prediction, Crosstabulation Summary Statistics.

Lung Feature	CXR Accuracy	P-Value	Sensitivity	Upper CL	Lower CL	Specificity	Upper CL	Lower CL
**Hilar lymphadenopathy**	60%	0.3557	55.6%	88.0%	23.1%	60.6%	48.8%	72.4%
**Cavity**	80%	0.0073	66.7%	100.0%	28.9%	81.2%	71.9%	90.4%
**Atelectasis**	19%	0.4549	16.7%	25.3%	8.1%	66.7%	13.3%	100.0%
**Pleural Effusion**	92%	<0.0001	50.0%	90.0%	10.0%	95.7%	90.8%	100.0%
**Nodule**	59%	0.0223	55.2%	67.1%	43.3%	87.5%	64.6%	100.0%

We also considered groupings for 63 relapse (Table 6) and 187 new (Table 7) patient cases. One might consider this patient case definition grouping as a surrogate for the duration of disease, and it seems reasonable to surmise that the longer a person has active TB the more lung damage may be present. We find differences between these two groups in terms of statistical significance for hilar lymphadenopathy and nodules. Lymphadenopathy was found in 7 of the 63 relapse patient cases (p = 0.0407) and 15 of the 187 new patient cases (p = 0.0006). Nodules were found in 61 of the relapse patient cases (p = 0.0669), and 166 of the new patient cases (p = 0.024).

**Table 6:** 63 TBPP CXR Annotations for Relapse TB Patient Case versus Qure.ai Binary Prediction, Crosstabulation Summary Statistics.

Lung Feature	CXR Accuracy	P-Value	Sensitivity	Upper CL	Lower CL	Specificity	Upper CL	Lower CL
**Hilar lymphadenopathy**	76%	0.0407	57.1%	93.8%	20.5%	78.6%	67.8%	89.3%
**Cavity**	75%	0.0001	90.9%	100.0%	73.9%	71.2%	58.8%	83.5%
**Atelectasis**	13%	0.641	9.8%	17.3%	2.4%	0.0%	0.0%	0.0%
**Pleural Effusion**	95%	0.0023	33.3%	86.7%	0.0%	98.3%	95.1%	100.0%
**Nodule**	65%	0.0669	63.9%	76.0%	51.9%	0.0%	0.0%	0.0%

**Table 7:** 187 TBPP CXR Annotations for New TB Patient Case versus Qure.ai Binary Prediction, Crosstabulation Summary Statistics.

Lung Feature	CXR Accuracy	P-Value	Sensitivity	Upper CL	Lower CL	Specificity	Upper CL	Lower CL
**Hilar lymphadenopathy**	71%	0.0006	73.3%	95.7%	51.0%	70.3%	63.5%	77.2%
**Cavity**	82%	<0.0001	69.2%	87.0%	51.5%	84.5%	78.9%	90.1%
**Atelectasis**	19%	0.9765	17.1%	22.6%	11.6%	83.3%	53.5%	100.0%
**Pleural Effusion**	94%	<0.0001	58.8%	82.2%	35.4%	97.1%	94.5%	99.6%
**Nodule**	57%	0.0237	54.8%	62.4%	47.2%	71.4%	52.1%	90.8%

**Fig 1:**
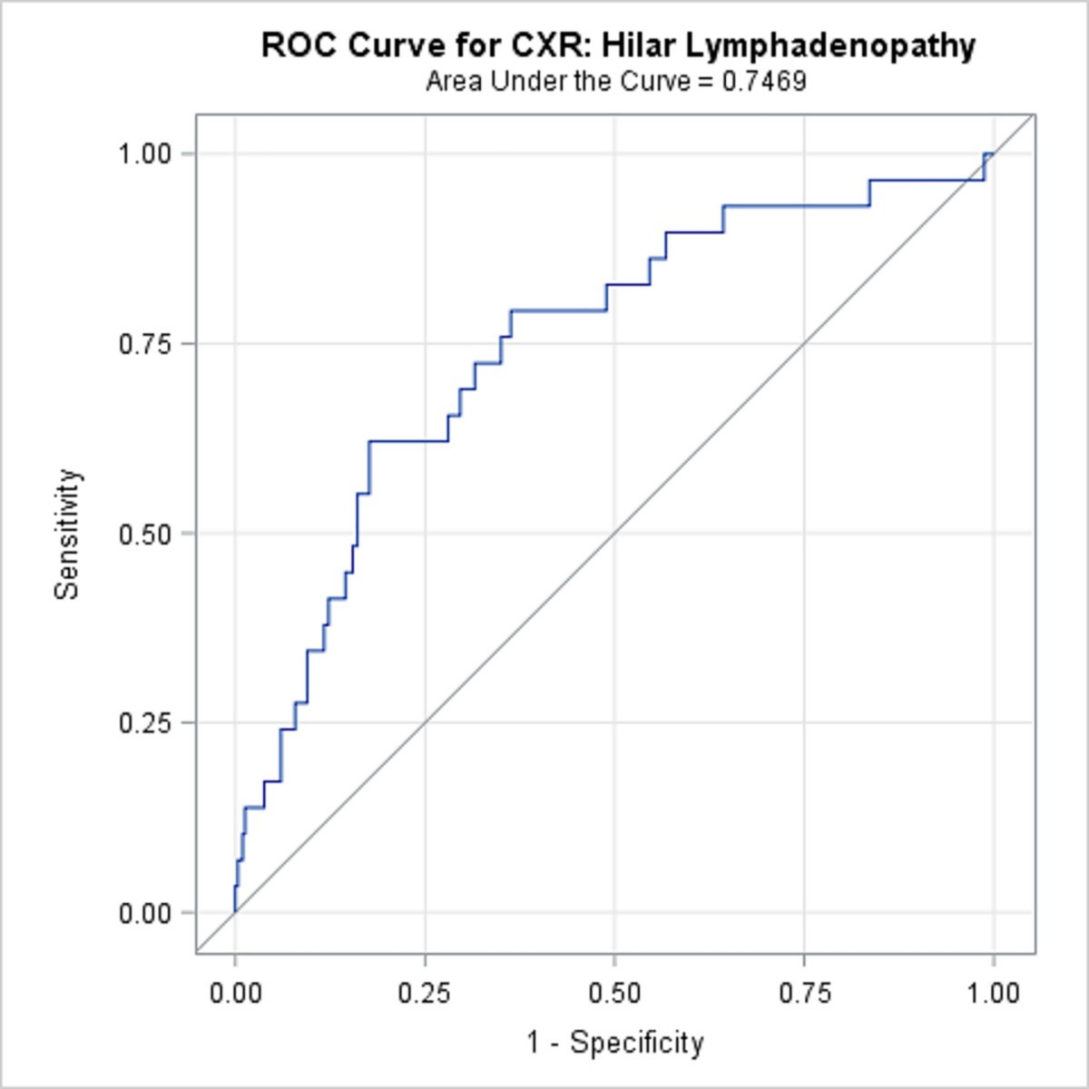
ROC Curve for CXR: Hilar Lymphadenopathy.

**Fig 2:**
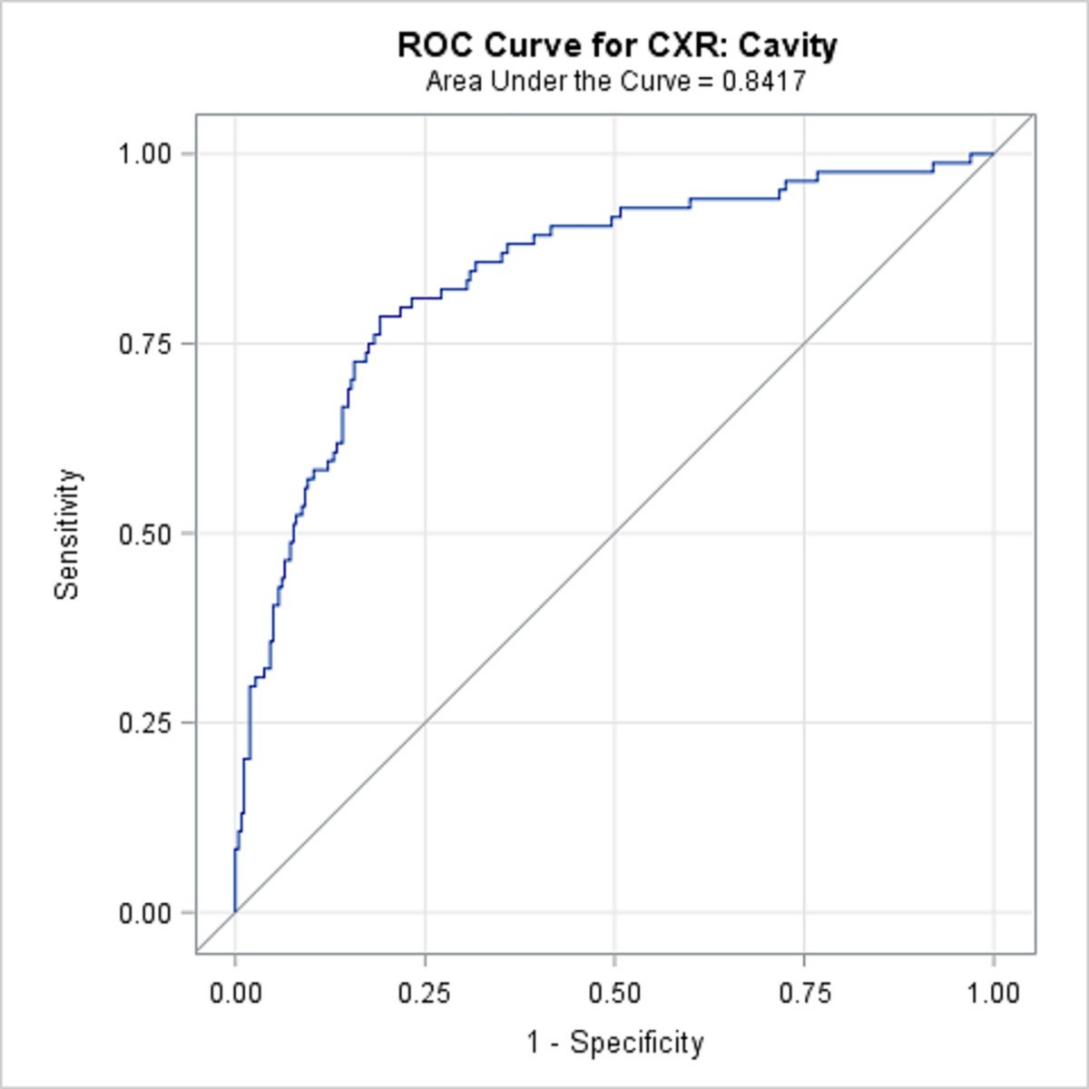
ROC Curve for CXR: Cavity.

**Fig 3:**
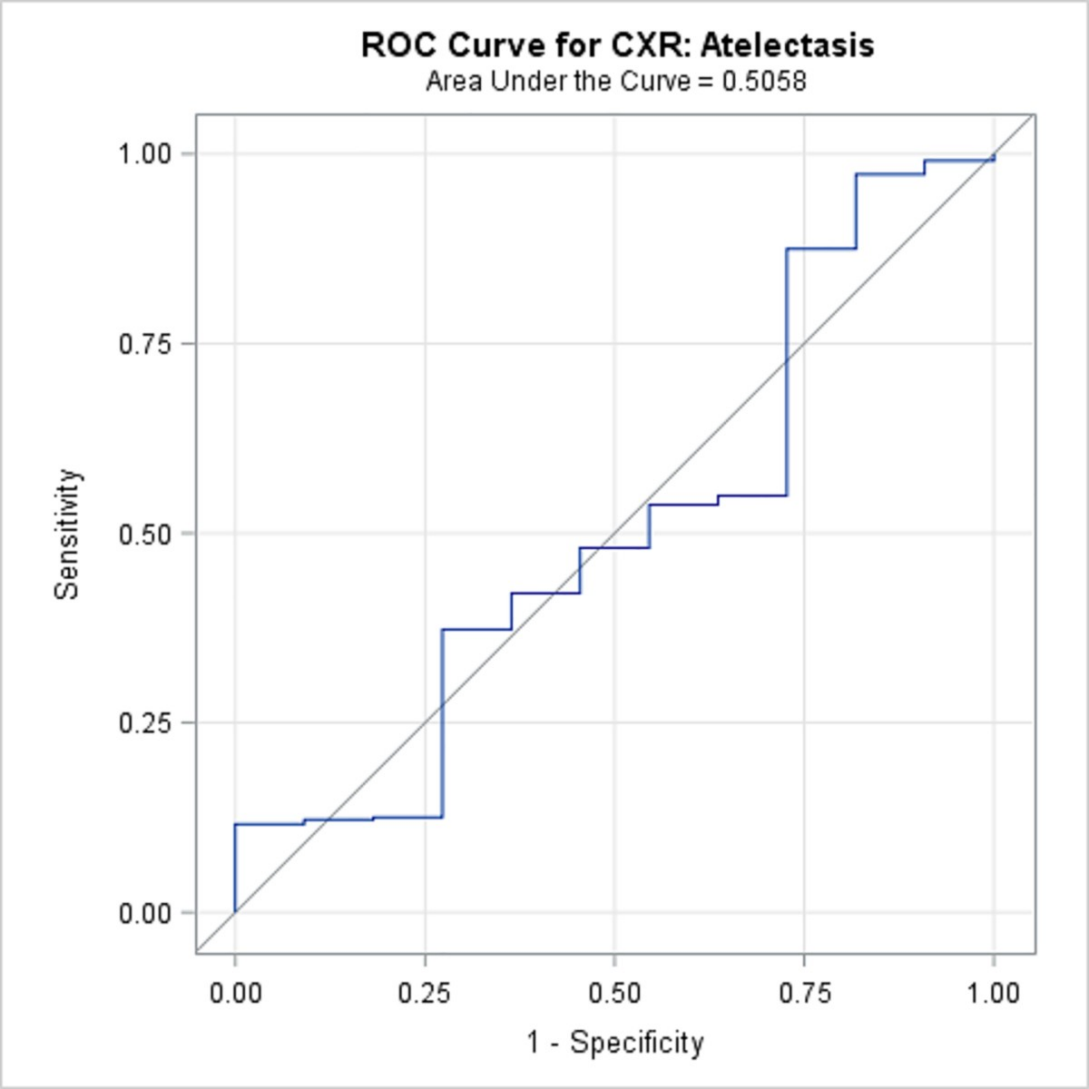
ROC Curve for CXR Atelectasis.

**Fig 4:**
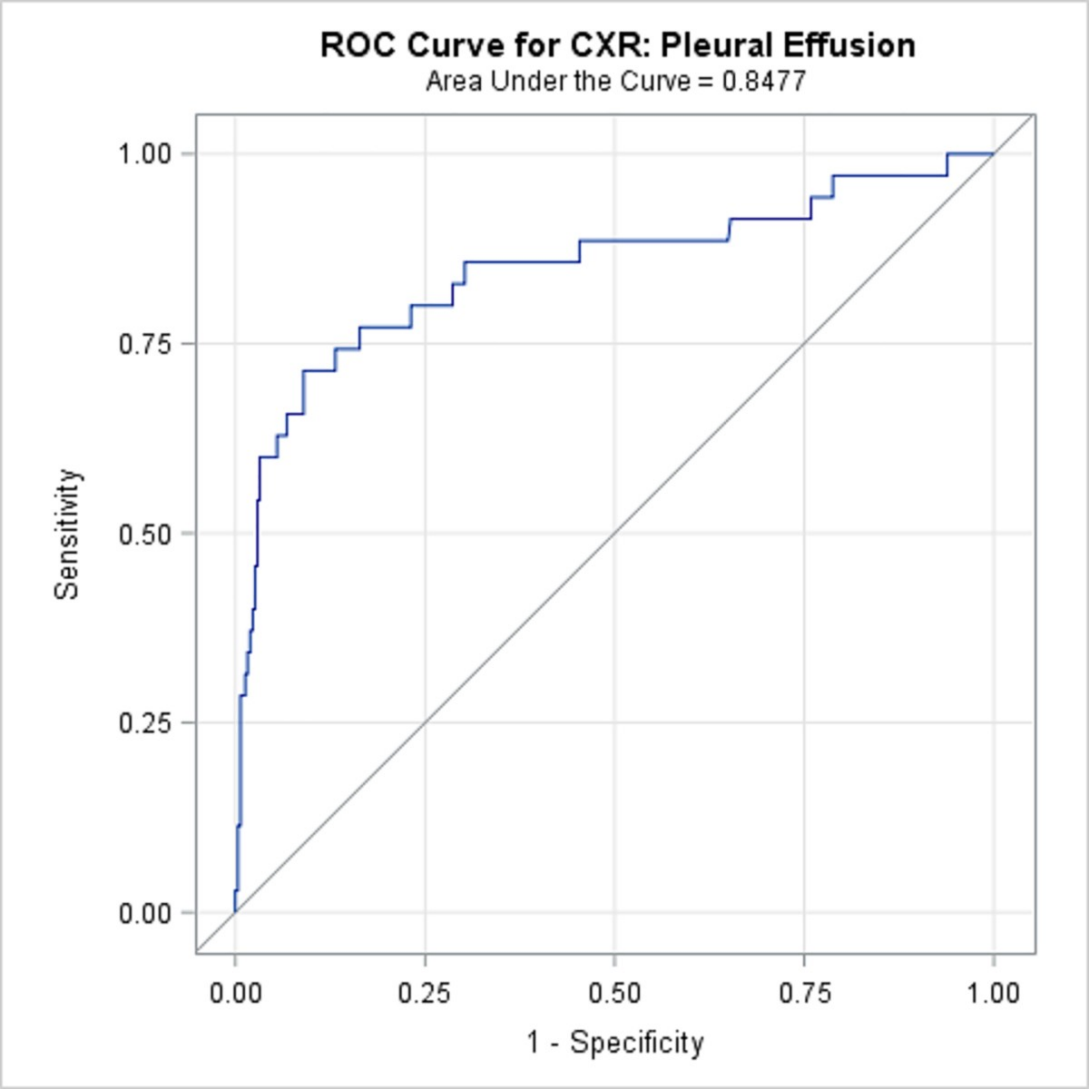
ROC Curve for CXR: Pleural Effusion.

**Fig 5:**
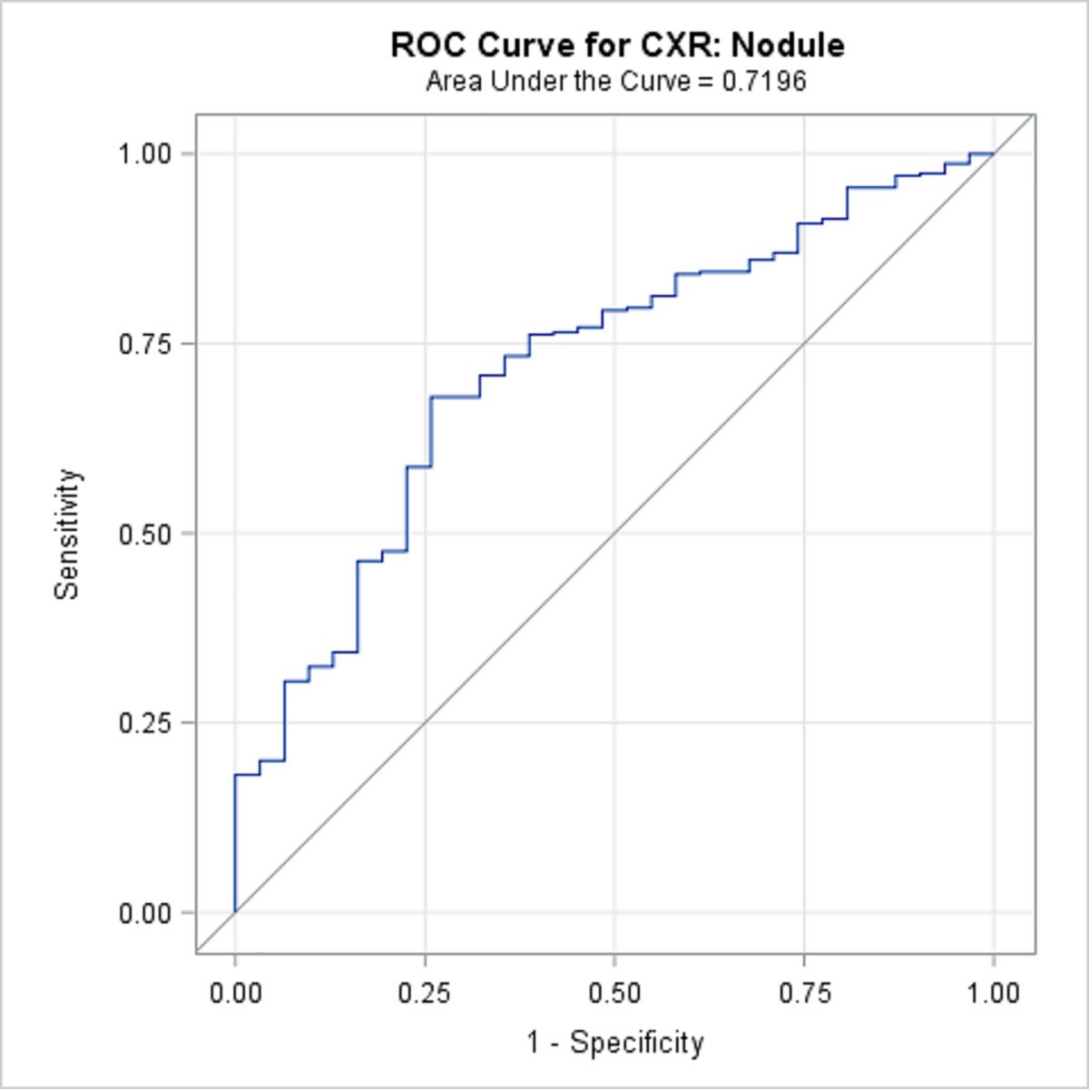
ROC Curve for CXR: Nodule.

TBPP annotations for the 346 CXRs compared with the Qure.ai continuous predicted score is summarized in Table 8 and illustrated with the receiver operating characteristic (ROC) curves in Figs 1–5. (Note the label “ROC Curve for CXR” in the title of the graphic.) The CXR Accuracy column in Table 8 indicates that the presence of pleural effusion and cavity are most accurate with a measure of 85% and 84% respectively, while atelectasis is least accurate at 51%. The accuracy measures are corroborated by the p-values indicated in Table 2 where cavity and pleural effusion were found to be of a higher statistical significance than atelectasis.

**Table 8:** 346 TBPP CXR Annotations versus Qure.ai Continuous Score, Receiver Operating Characteristic Statistics Summary.

Lung Feature	CXR Accuracy	95% Wald Confidence Limits	
**Hilar lymphadenopathy**	74.69%	65.0%	84.4%
**Cavity**	84.17%	79.1%	89.3%
**Atelectasis**	50.58%	31.1%	70.1%
**Pleural Effusion**	84.77%	76.2%	93.3%
**Nodule**	71.96%	62.9%	81.0%

**Fig 6:**
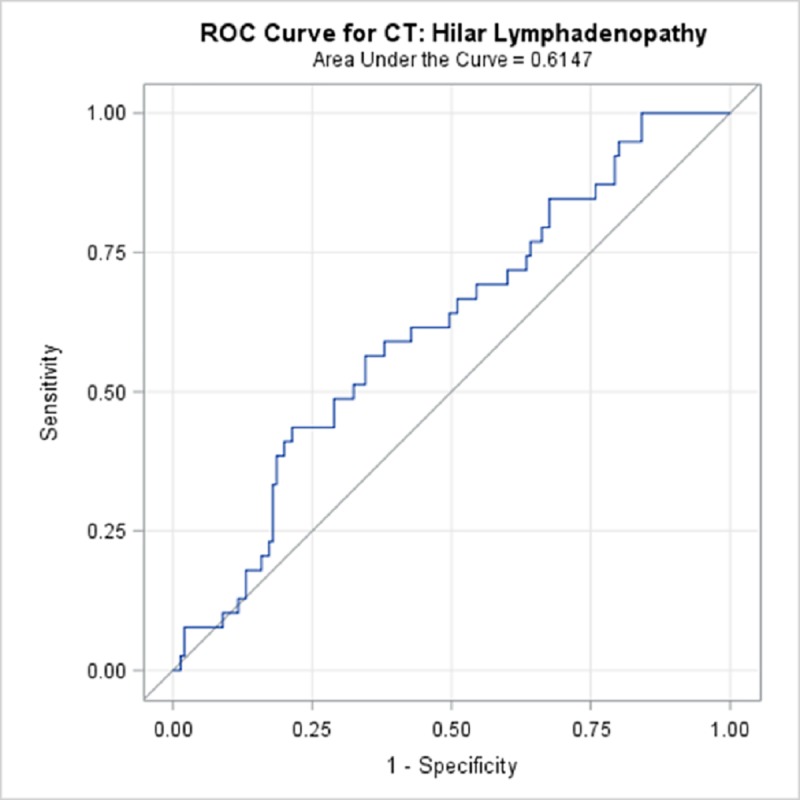
ROC Curve for CT: Hilar Lymphadenopathy.

**Fig 7:**
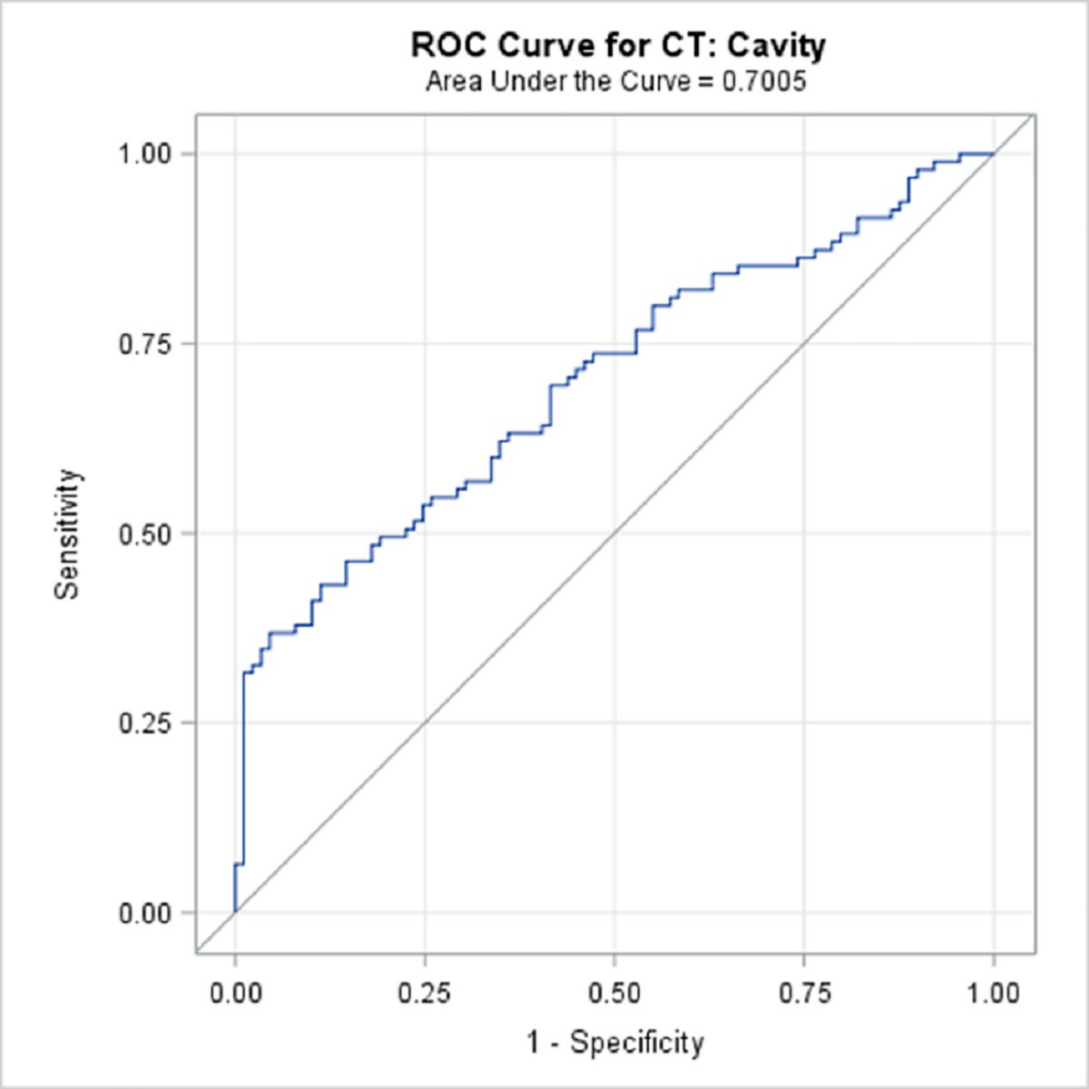
ROC Curve for CT: Cavity.

**Fig 8:**
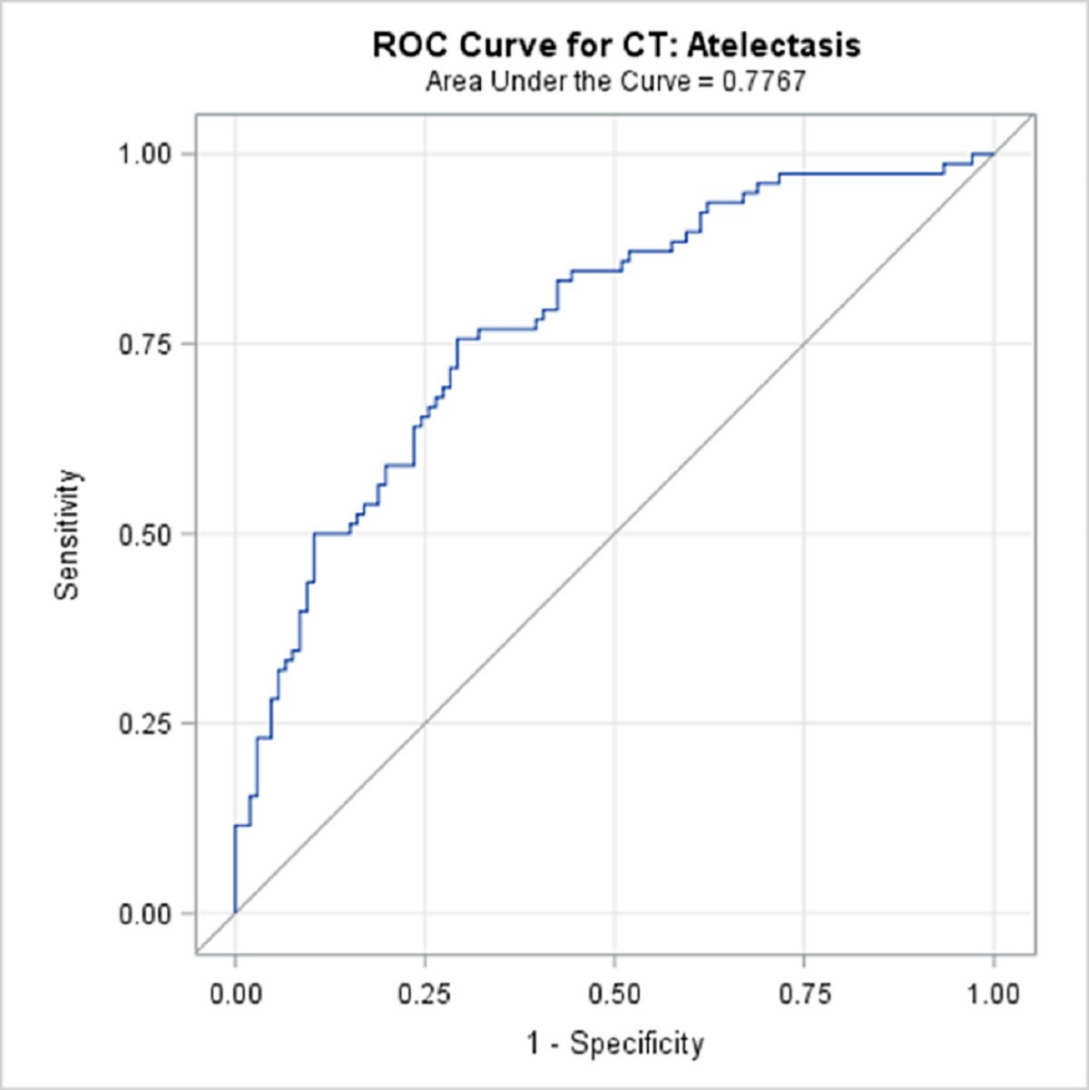
ROC Curve for CT: Atelectasis.

**Fig 9:**
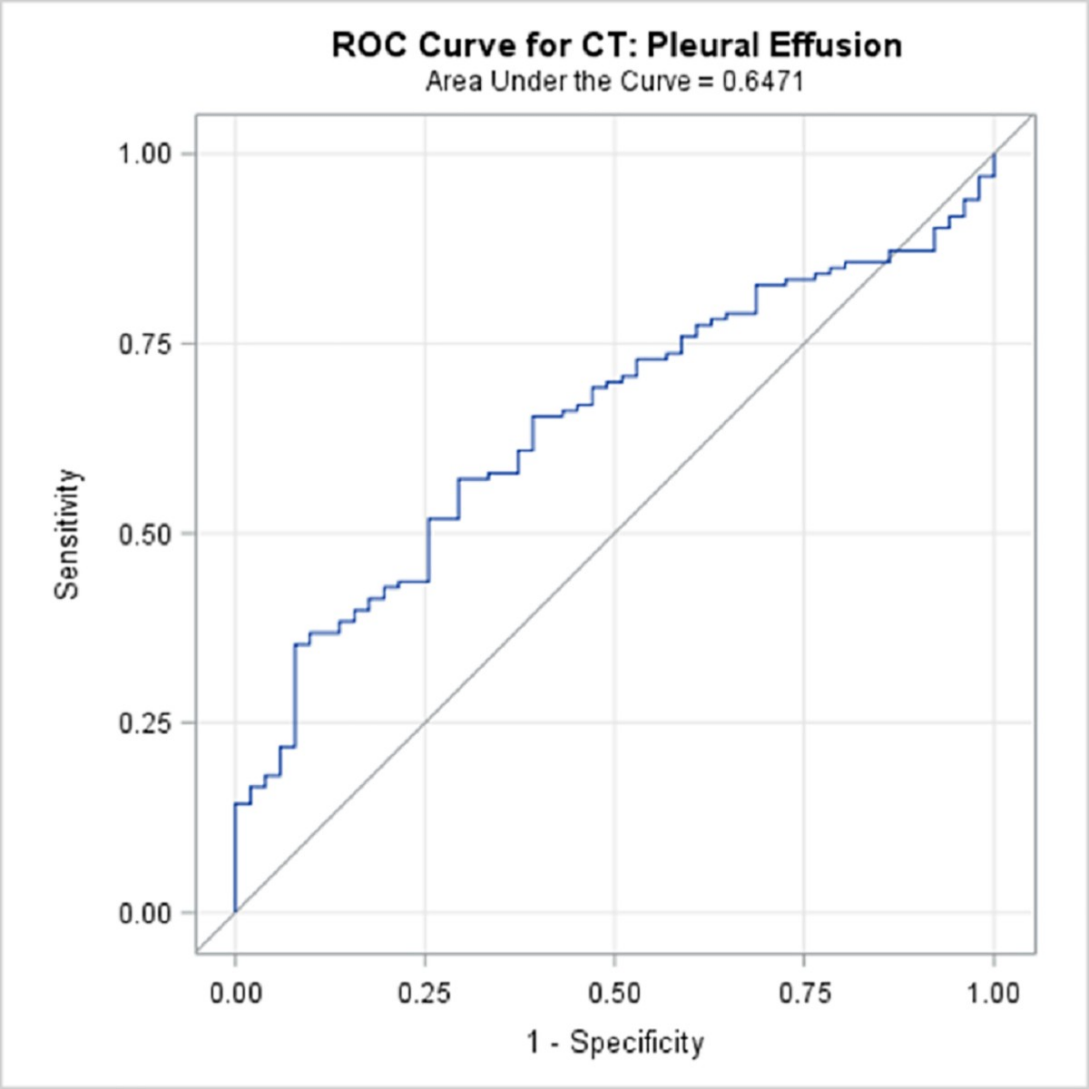
ROC Curve for CT: Pleural Effusion.

**Fig 10:**
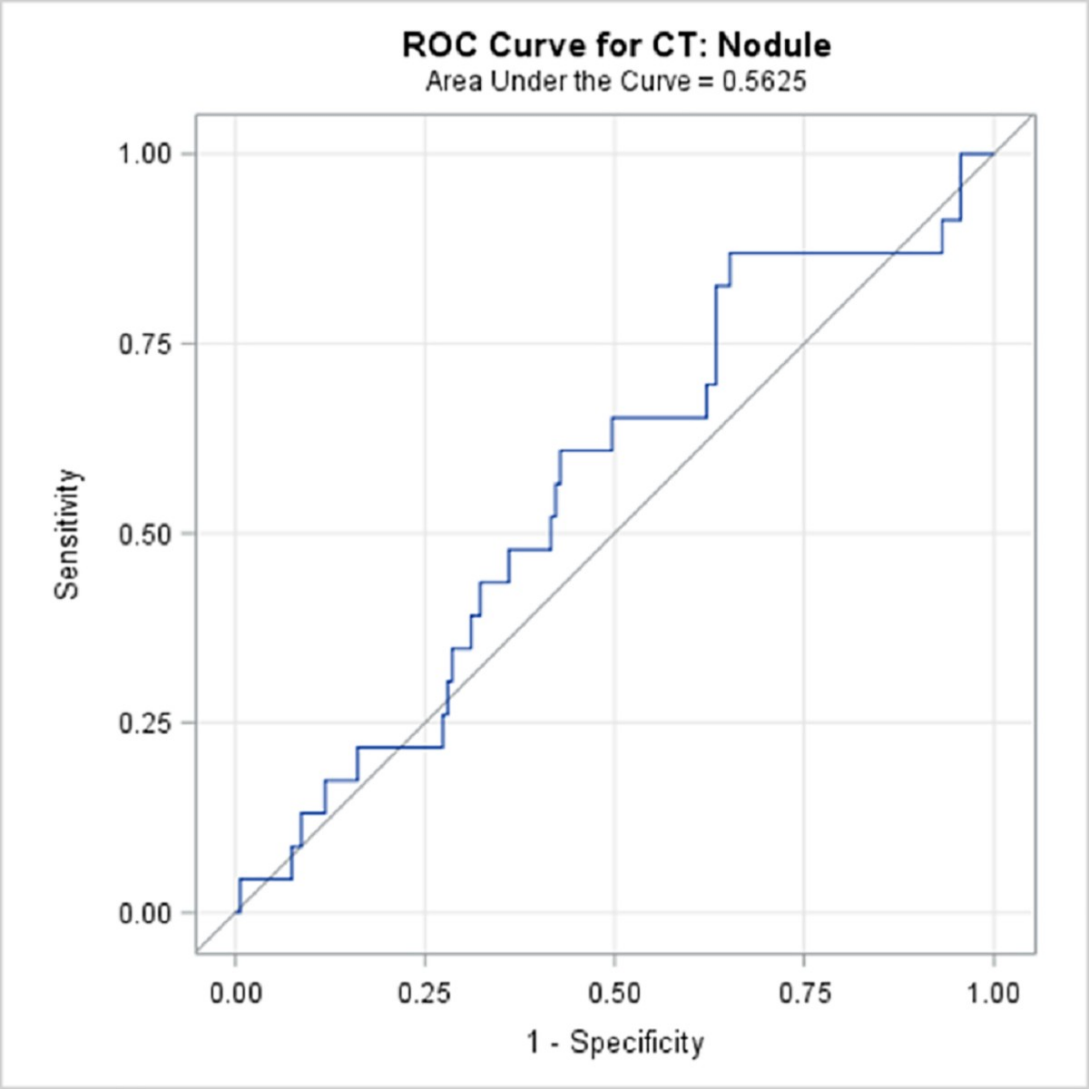
ROC Curve for CT: Nodule.

TBPP annotations for the 184 CTs compared with the Qure.ai continuous predicted score is summarized in Table 9 and illustrated with the receiver operating characteristic (ROC) curves in Figs 6–10. (Note the label “ROC Curve for CT” in the title of the graphic.) The CT Accuracy column in Table 9 indicates that atelectasis and cavity are the most accurate with a measure of 78% and 70% respectively, and nodule is least accurate at 56%. The accuracy measures are corroborated by the p-values indicated in Table 3 where atelectasis and cavity were found to be of a higher statistical significance than nodule.

**Table 9:** 184 TBPP CT Annotations versus Qure.ai Continuous Score, Receiver Operating Characteristic Statistics Summary.

Lung Feature	CT Accuracy	95% Wald Confidence Limits	
**Hilar lymphadenopathy**	61.47%	51.8%	71.1%
**Cavity**	70.05%	62.5%	77.6%
**Atelectasis**	77.67%	70.9%	84.4%
**Pleural Effusion**	64.71%	56.5%	72.9%
**Nodule**	56.25%	43.9%	68.6%

For the purpose of examining the potential value of Qure.ai classifiers as predictors of disease outcome, the patient case cohorts consist of 220 (78%) patients with an outcome of cured, and 61 (22%) with an outcome of died or treatment failure. For these 281 patient cases, the type of resistance distribution is 69 (24.5%) extensively drug resistant, 163 (58.1%) MDR or mono drug resistant, and 49 (17.4%) drug sensitive. The patient case definition distribution is 144 (51.2%) new, 61 (21.7%) failure, 41 (14.6%) relapse, 32 (11.4%) treatment after default, and 3 (1.1%) other.

Table 10 introduces the Qure.ai classifier binary values (0 or 1) indicating the presence of the lung feature. The summary statistics compare the Qure.ai classifier to the binary outcome groups for cured versus died/failure. With p-value less than .05, there is a statistically significant relationship for nodule, hilar lymphadenopathy, cavity, consolidation, and fibrosis.

**Table 10:** 281 Qure.ai classifier binary value predicting TBPP outcome died/failure.

Lung Feature	Number of cases present	Fisher's Exact Test *P*-value	Sensitivity	Specificity
**Abnormal CXR**	263	0.770	95.1%	6.8%
**Blunted CP Angle**	126	0.059	55.7%	58.2%
**Cardiomegaly**	16	0.536	3.3%	93.6%
**Pleural Effusion**	38	0.056	21.3%	88.6%
**Nodule**	174	0.001	80.3%	43.2%
**Hilar lymphadenopathy**	93	0.046	44.3%	70.0%
**Cavity**	120	0.002	60.7%	62.3%
**Opacity**	249	0.108	95.1%	13.2%
**Atelectasis**	82	0.204	36.1%	72.7%
**Consolidation**	179	0.035	75.4%	39.5%
**Indicator of TB disease**	242	0.208	91.8%	15.5%
**Fibrosis**	208	0.003	88.5%	30.0%

The significance of differences between average scores of the Qure.ai classifiers were examined between the two cohorts using the nonparametric WMW test. As shown in Table 11, all lung features except blunted costophrenic angle (CP) angle and cardiomegaly are statistically significantly different at the P-value < .05 level. The significant lung features are as follows: abnormal CXR (p = 0.0005), pleural effusion (p = 0.048), nodule (p = 0.0004), hilar lymphadenopathy (p = 0.0038), cavity (p = 0.0002), opacity (p = 0.0006), atelectasis (p = 0.0074), consolidation (p = 0.0004), indicator of TB disease (p = < .0001), and fibrosis (p = < .0001). This test allows us to conclude that the distribution of scores for these features are not equally distributed between the cohort that was cured versus the cohort that died or experienced treatment failure.

**Table 11:** 281 Qure.ai continuous classifier score predicting TBPP outcome.

	Cured: Mean Score	Died/Failure: Mean Score	WMW Test *P*-values
**Abnormal CXR**	132.05	173.28	0.0005
**Blunted CP Angle**	136.75	156.31	0.0963
**Cardiomegaly**	137.72	152.84	0.1999
**Pleural Effusion**	135.93	159.29	0.048
**Nodule**	131.84	174.03	0.0004
**Hilar lymphadenopathy**	133.54	167.88	0.0038
**Cavity**	131.35	175.77	0.0002
**Opacity**	132.08	173.16	0.0006
**Atelectasis**	134.11	164.85	0.0074
**Consolidation**	131.82	174.1	0.0004
**Indicator of TB disease**	130.28	179.67	< .0001
**Fibrosis**	130.53	178.75	< .0001

These observed statistically significant results comparing the distribution of Qure.ai scores within the two outcome groups motivates a closer examination using the receiver operating characteristic (ROC) curves. The ROC curve statistics are summarized in Table 12.

**Table 12:** Qure.ai classifier score predicting TBPP outcome died; receiver operating characteristic statistics summary.

Lung Feature	Accuracy	95% Wald Confidence Limits	
**Abnormal CXR**	64.67%	57.1%	72.2%
**Blunted CP Angle**	56.96%	49.0%	64.9%
**Cardiomegaly**	55.38%	47.4%	63.4%
**Pleural Effusion**	58.32%	49.7%	66.9%
**Nodule**	65.01%	57.6%	72.4%
**Hilar lymphadenopathy**	62.22%	54.4%	70.0%
**Cavity**	65.80%	58.1%	73.5%
**Opacity**	64.62%	57.0%	72.2%
**Atelectasis**	61.30%	53.9%	68.7%
**Consolidation**	65.04%	57.5%	72.6%
**Indicator of TB disease**	67.58%	59.8%	75.3%
**Fibrosis**	67.16%	59.4%	74.9%

The Accuracy column in Table 12 details that the indicator of TB disease and fibrosis were most accurate with a measure of 68% and 67% respectively, while costophrenic angle (CP) angle and cardiomegaly are least accurate with measures of 55% and 57%. These accuracy measures are corroborated by the p-values indicated in Table 11 where indicator of TB disease, and fibrosis were found to be of a higher statistical significance than blunted costophrenic angle (CP) angle and cardiomegaly.

## Discussion

Changes in the lungs are combined effects of host response to the Mycobacterium tuberculosis (M.tb) pathogen invasion and to the regimen drugs [28]. As part of TB Portals program, we engage in testing new prediction methods against TBPP database of patient CXRs and CTs, searching for the presence of reliable correlations with the progression of TB disease and, ultimately, to treatment outcome [14, 29]. We have demonstrated that the results of fully automated Qure.ai CXR classifiers for CXRs from TB patient case data were consistent with annotations by radiologists. The important distinction worth re-iterating here is that TBPP database had specifically targeted drug-resistant tuberculosis, and many CXRs for test dataset came from DR-TB patients. Treatment for DR-TB is more expensive and less efficient, therefore any improvements in diagnostics and monitoring are highly desirable. We were encouraged to find that Qure.ai classifiers add meaningful insight into TB disease prognosis and treatment outcome, and therefore are good candidates for fast, accurate, uniform, large-scale assistance when and where there is a shortage of radiologists.

For every computer-based classifier, the development begins with as large a collection of training data as possible containing sufficient number of records that represent the observation we wish to classify [30]. The development cycle ends with a validation, or tuning step. During this step the model is initially evaluated, and parameters are changed based on data that was not included in the initial classifier training. One common challenge with increasingly popular (due to their performance in many scientific and business analysis tasks) deep learning algorithms is the element of “black box”. It is challenging for a human to understand how a deep learning algorithm arrives at the classification. This is still an active area of research [31].

The final step in classifier development is testing aimed at quantifying the accuracy and effectiveness of the classifier. The identification of true positive observations for CXR annotations is a widely known challenge. For CXR annotation classifiers, within TB and for other disease targets, some evaluation test sets are created using the consensus of expert raters [32]. As in TBPP, many regions, i.e. patient populations around the world, will not have this luxury. In this study we attempt to address the challenge by leveraging the Radiologist annotations using CT images, assuming that the Radiologist’s accuracy may increase given the additional detailed image quality. We’ve shown that the results between Radiologist annotations of CXR and CTs revealed consistent performance of the Qure.ai classifiers.

A unique challenge in applying deep learning classifiers to novel data, including novel testing data, is that the classifiers may behave as a step function [33]. Meaning that for some input data that was not in the training set for the classifier, the classifier will generate an incorrect predicted result that would be immediately recognizable to a human. Examples of these types of errors may be widely reported [34]. This is important, as classifiers may demonstrate very strong results against a testing sample that shares many of the same attributes as the training and validation data, and yet fail when challenged with novel data.

Testing and validating relevant artificial intelligence algorithms (both machine learning and deep learning classifiers) in research settings, such as TBPP, is critically important toward progressing to a common understanding and confidence in using these standardized, repeatable measures. Expanding the size, and as importantly, the number of representative patient case features within the training and testing databases will remain important to artificial intelligence algorithm development, validation, and testing outcomes. Considering our study objectives, the single Qure.ai TB screening classifier was previously tested so we did not aim to re-test it using the predominately DR-TB TBPP images that might be presumed to have more pronounced lung features indicating TB disease. We specifically examined Qure.ai deep learning classifiers used for CXR annotation developed–trained and validated—in India, a country population experiencing a lower burden of DR-TB, using CXRs collected from TBPP member countries experiencing a high burden of DR-TB [35].

In this analysis we noted that Qure.ai classifiers are related to patient case outcomes; i.e. higher classifier scores are related to poorer outcomes. These findings may help in uniform screening and patient monitoring of CXRs to identify the most problematic patient cases that require additional scrutiny for both diagnosis and treatment.

The TBPP is active and growing, currently collecting and curating TB patient case data from diverse sources and multiple medical domains, including ten country sites. TBPP offers a unique opportunity to address issues with fully utilizing radiological imaging to further TB research. By collecting and curating data from diverse sources and multiple medical domains in ever-increasing depth and breadth the TBPP offers the value of big data that enables the reusability of data, in conformity with the NIH’s Findable, Accessible, Interoperable, and Reusable (FAIR) principles [36, 37]. Here we have leveraged these data to assess the Qure.ai machine learning classifiers and demonstrated that the collection is statistically significantly related to radiology annotations that are markers of TB disease.

TBPP has an emphasis on collecting the rare, atypical and most dangerous TB cases through a global natural history study. Of the 1,425 physician-validated publicly available patient cases, 1,179 (83%) are drug resistant. Hence, TBPP offers a valuable resource for confirming deep learning algorithms against this segment of the overall patient population. The analysis results offer some signal that additional CXR image collection may be warranted to account for the differences among drug resistant patient cases and those with more advance TB disease as input to deep learning classifiers.

The TBPP imaging database has previously shown itself useful to machine learning algorithm development [14, 29]. Expanding upon and improving these initiatives is a key TBPP goal. Continued application and testing using deep learning classifiers is important as TBPP seeks to expand and further improve MDR-TB patient diagnostics and outcomes. The authors invite other researchers with artificial intelligence algorithms useful to combat TB to collaborate with TBPP.

Some caution is warranted here. The Qure.ai classifiers can aid in the interpretation of CXR findings and is useful for examining changes over time [23]. However, a list of twelve classifiers, the current offering, is unlikely to replace a radiologist due to limited specificity for categorizing specific findings–which are much more numerous. Also, considering the CXR automated annotation classifiers that are not TB specific, we cannot assume that classifiers developed primarily from non-TB patient images can accurately characterize those with TB disease. Using a tested, consistent, uniform measure across a database for research purposes is different than using it for a specific patient case.

The use of machine learning and deep learning tools in the field of health care is becoming increasingly common. The need to discriminate between model implementations and to test these models in various scenarios is also increasing. For radiological assessment, these tools, if shown to be representative across the spectrum of imaging machines and image collection methods, as well as TB patient lung characteristics influenced by factors such as pathogen strain, ethnicity, gender and socio-economics, etc. offer a standard, consistent and repeatable measure. This is particularly valuable, as there is a lack of a global standard for chest X-ray feature annotation, a paucity of radiologists, and a need for cost-effective tools in poor countries that are the most burdened with TB disease.

## Conclusion

We demonstrated that the Qure.ai qXR CXR annotations for cavity, nodule, pleural effusion, and hilar lymphadenopathy, are in statistical agreement with radiologist CXR annotations. A corresponding analysis of patient case matched CT image annotations recorded within 30 days of a CXR supports this result, demonstrating statistical significance for cavity, pleural effusion, hilar lymphadenopathy, and atelectasis. In addition, the Qure.ai qXR CXR annotations for abnormal CXR, blunted CP angle, cardiomegaly, pleural effusion, nodule, hilar lymphadenopathy, cavity, opacity, atelectasis, consolidation, indicator of TB disease, and fibrosis are related to TB patient case outcomes. Hence, these new lung feature descriptors resulting from use of the Qure.ai qXR product’s CXR classification annotations are useful for fast, accurate, uniform, large-scale assistance.

Addressing the growing threat of DR-TB requires a comprehensive understanding of the disease, which could be achieved by multi-center, global collaborations contributing data, algorithms and classifiers from multiple domains. The TB Portals Program (TBPP, https://TBPortals.niaid.nih.gov/) was established with this mission in mind, consolidating curated and de-identified patient socioeconomic, clinical, radiological, and genomic information from TB cases.

## Supporting information

S1 Source Data TB Portals Program source data files in comma separated value (CSV) format. (ZIP)
